# Supplementary material for: Spatiotemporal Variation of Urban Plant Diversity and above Ground Biomass in Haikou, China
Source: Biology (Basel). 2022 Dec 14;11(12):1824. doi: 10.3390/biology11121824 (PMC9775028; doi:10.3390/biology11121824)

Table S1. Mean value and standard deviation of AGB and the number of total, tree, shrub and herb species in primary UFUs in Haikou in 2015 and 2021.

Primary UFU type	AGB		Number of tree species		Number of shrub species		Number of herb species		Number of total species	
	2015	2021	2015	2021	2015	2021	2015	2021	2015	2021
Public affairs service districts	$2.07\times10^{7} \pm 1.61\times10^{7}$	$4.78\times10^{7} \pm 4.61\times10^{7}$	3.91 ± 1.95	5.90 ± 2.84	1.43 ± 0.58	5.64 ± 2.36	2.48 ± 1.79	7.92 ± 4.98	7.27 ± 2.48	19.20 ± 8.01
Industry and business districts	$2.83\times10^{7} \pm 2.68\times10^{7}$	$4.12\times10^{7} \pm 3.50\times10^{7}$	3.59 ± 1.73	5.65 ± 2.46	1.34 ± 0.57	5.73 ± 2.03	1.58 ± 0.83	7.35 ± 3.87	6.52 ± 2.23	18.73 ± 5.93
Residential districts	$1.77\times10^{7} \pm 1.52\times10^{7}$	$2.78\times10^{7} \pm 2.17\times10^{7}$	4.38 ± 2.62	5.28 ± 2.17	1.48 ± 0.55	5.61 ± 2.83	2.02 ± 1.16	6.50 ± 4.07	7.87 ± 3.10	17.38 ± 7.39
Recreation and leisure districts	$1.01\times10^{8} \pm 7.80\times10^{7}$	$4.86\times10^{7} \pm 4.88\times10^{7}$	3.61 ± 1.52	5.5 ± 2.51	2.12 ± 1.46	4.9 ± 3.11	4.48 ± 2.96	3.44 ± 1.46	10.21 ± 4.02	19.30 ± 7.55
Transportation	$2.12\times10^{7} \pm 1.60\times10^{7}$	$4.43\times10^{7} \pm 6.05\times10^{7}$	3.33 ± 3.82	6.13 ± 2.80	1.12 ± 0.63	6.08 ± 2.90	1.14 ± 0.87	8.04 ± 4.80	5.58 ± 2.93	20.25 ± 7.91
Undeveloped land	0 ± 0	$1.88\times10^{7} \pm 1.44\times10^{7}$	3.28 ± 0	5 ± 1.41	0 ± 0	5.5 ± 0.70	0 ± 0	3.44 ± 1.46	0 ± 0	22.5 ± 4.5

Table S2. Mean value and standard deviation of the AGB and the number of trees, shrubs and herbs in secondary UFUs in Haikou

Secondary UFU type	AGB		Number of trees		Number of shrubs		Number of herbs	
	2015	2021	2015	2021	2015	2021	2015	2021
Governmental agencies	$1.86\times10^{6} \pm 7.51\times10^{5}$	$4.37\times10^{6} \pm 6.21\times10^{6}$	3.42 ± 2.02	5.33 ± 2.46	1.35 ± 0.45	6.07 ± 2.40	1.53 ± 1.08	10.25 ± 4.90
Colleges or universities	$2.91\times10^{6} \pm 2.51\times10^{5}$	$3.20\times10^{6} \pm 3.03\times10^{6}$	4.60 ± 1.52	4.00 ± 1.58	1.39 ± 0.38	4.20 ± 2.59	1.93 ± 0.61	5.00 ± 2.91
Primary and middle schools	$2.06\times10^{6} \pm 1.76\times10^{6}$	$6.14\times10^{6} \pm 4.88\times10^{6}$	3.22 ± 1.39	7.44 ± 2.65	1.22 ± 0.35	6.30 ± 2.71	2.18 ± 0.86	8.44 ± 4.75
Research institutes	$3.71\times10^{6} \pm 0$	$6.80\times10^{6} \pm 0$	2.50 ± 3.54	3 ± 4.24	0.60 ± 0.85	4.00 ± 5.66	1.67 ± 2.35	5.50 ± 7.78
Hospitals	$1.70\times10^{6} \pm 1.37\times10^{6}$	$2.72\times10^{6} \pm 1.97\times10^{6}$	4.78 ± 2.39	5.78 ± 3.73	1.80 ± 0.87	4.78 ± 1.39	2.04 ± 0.70	4.44 ± 4.67
Industry	$4.27\times10^{6} \pm 4.03\times10^{6}$	$3.32\times10^{6} \pm 2.24\times10^{6}$	2.79 ± 2.04	6.00 ± 3.11	0.96 ± 0.47	6.43 ± 1.13	1.27 ± 0.68	9.00 ± 4.58
Hotels	$2.28\times10^{6} \pm 1.67\times10^{6}$	$2.06\times10^{6} \pm 1.76\times10^{6}$	4.00 ± 2.12	4.78 ± 2.54	1.41 ± 0.48	5.44 ± 2.70	1.64 ± 0.82	6.89 ± 4.78
Industrial offices	$1.81\times10^{6} \pm 1.47\times10^{6}$	$4.53\times10^{6} \pm 4.35\times10^{6}$	3.88 ± 0.99	6.38 ± 1.92	1.37 ± 0.41	5.38 ± 2.13	1.94 ± 0.83	6.25 ± 3.06
Supermarkets	$4.28\times10^{6} \pm 6.53\times10^{5}$	$6.32\times10^{6} \pm 4.81\times10^{6}$	3.5 ± 0.71	5.00 ± 2.53	2.25 ± 1.06	6.00 ± 0	1.00 ± 1.41	8.00 ± 4.24
Low-density residential areas	$7.35\times10^{5} \pm 2.53\times10^{5}$	$2.34\times10^{6} \pm 5.69\times10^{5}$	3.00 ± 2.65	4.00 ± 1.00	1.89 ± 1.54	4.33 ± 2.08	2.00 ± 1.33	7.67 ± 6.56
High-density residential areas	$1.88\times10^{6} \pm 1.56\times10$	$2.83\times10^{6} \pm 2.27\times10^{6}$	4.52 ± 2.63	5.17 ± 2.07	1.44 ± 0.38	5.73 ± 2.89	2.02 ± 1.67	6.40 ± 4.00
Parks	$2.87\times10^{7} \pm 2.17\times10^{7}$	$7.01\times10^{6} \pm 5.28\times10^{6}$	3.58 ± 0.81	5.50 ± 2.12	2.11 ± 0.43	5.00 ± 3.10	5.00 ± 1.60	9.67 ± 5.16
Museums	$7.51\times10^{6} \pm 5.99\times10^{6}$	$2.59\times10^{6} \pm 2.21\times10^{6}$	3.65 ± 2.42	6.25 ± 2.63	2.15 ± 2.51	4.75 ± 3.59	3.70 ± 4.01	7.75 ± 6.29
Bus parking	$2.92\times10^{6} \pm 1.36\times10^{6}$	$6.97\times10^{6} \pm 7.07\times10^{6}$	8.00 ± 5.29	5.00 ± 4.36	0.83 ± 0.76	5.25 ± 3.20	0.89 ± 1.02	4.66 ± 2.88
Main roads and secondary roads	$2.01\times10^{6} \pm 1.61\times10^{6}$	$4.02\times10^{6} \pm 5.76\times10^{6}$	2.63 ± 1.51	6.40 ± 2.64	1.14 ± 0.62	6.25 ± 2.90	1.17 ± 0.87	8.8 ± 4.82
Wetland	0 ± 0	$1.88\times10^{6} \pm 1.45\times10^{6}$	0 ± 0	5.14 ± 2.12	0 ± 0	4.00 ± 5.66	0 ± 0	12.00 ± 4.24

Figure S1. Boxplots of AGB and the number of total, tree, shrub and herb plant species in secondary UFUs in 2015 and 2021.

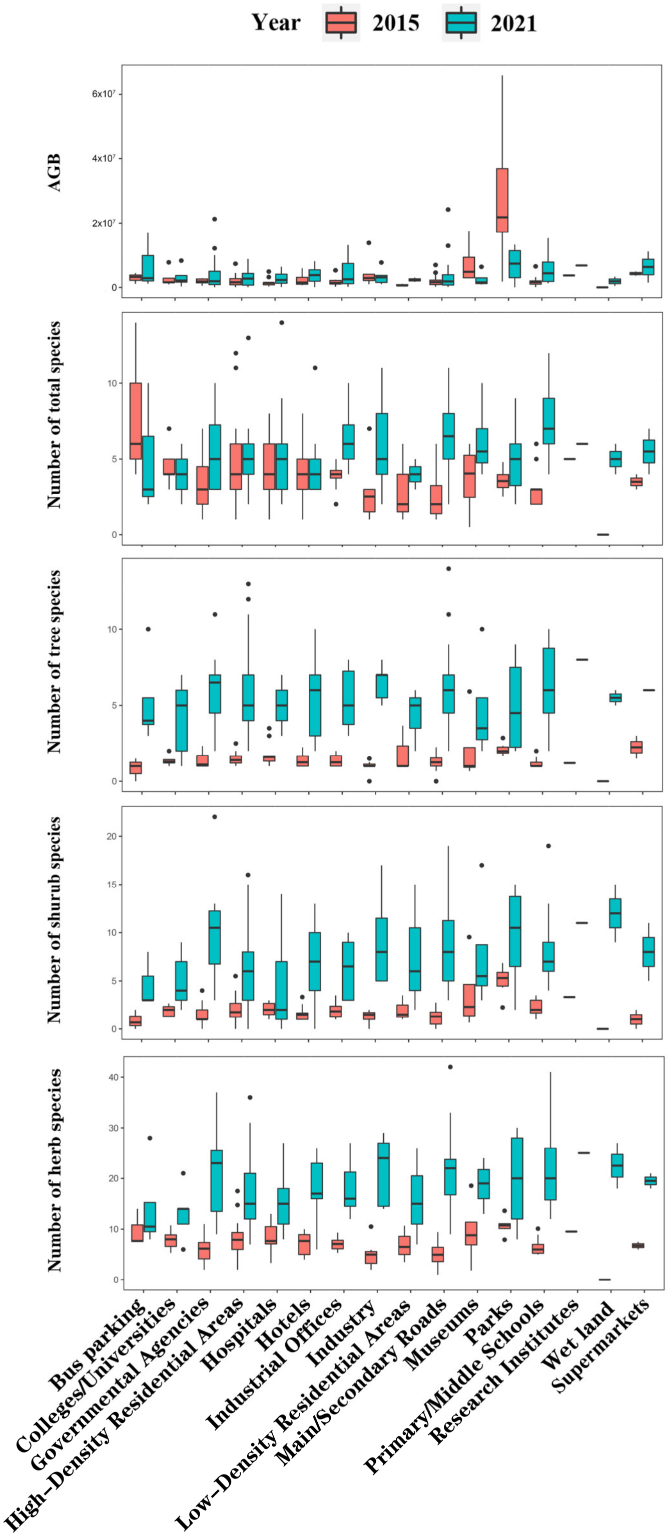

Supplement: Supplementary file 1 [file biology-11-01824-s001.zip › biology-2053662-Supplementary Materials-New Version.pdf]
